# Aggression, autoaggression and stress in people with a heart attack

**DOI:** 10.1192/j.eurpsy.2025.1961

**Published:** 2025-08-26

**Authors:** V. Jovanovska, K. Petkovska

**Affiliations:** 1 Psychiatry, PHI General Hospital Kumanovo, Kumanovo; 2 PMI Nuhi Jusufi, Kumanov, North Macedonia

## Abstract

**Introduction:**

A human represents wholeness of biological, psychological and social nature and below impact of psychosocial stress preserves the homeostasis of the organism manifests a complete biopsychosocial response is therefore necessary in professional practice to be taken psychoneuroendocrine immunological approach in treating certain somatic and psychological.

**Objectives:**

Determining emotions, various types of aggression in people who have experienced a heart attack and is currently under the influence of chronic psychosocial stress of moderate intensity.

**Methods:**

The examination was conducted on an outpatient basis on 14 subjects aged 51 to 72. age of male and female who experienced a heart attack. Applied: Azingerova aggression scale, PSQ stress test, Zung anxiety scale, Zung depression scale.

**Results:**

The respondents show a stress reaction at the level of moderate stress (30-60) with dominant feelings of: fatigue, tension, dissatisfaction, overload. Anxious symptoms are at the level of mild to moderate (45-59). Depressive symptoms are at the mild level (50-59). They have higher values on the aggression test. In both sexes (male and female) they have the highest values in self-aggression. Somewhat lower is verbal aggression while emotional aggression has average values. In the female gender, aggression is the lowest through physical force, while the use of aggression through physical force is occasional for men. Both sexes show little aggression towards objects around them.

**Conclusions:**

In people with somatic diseases, in the case of a heart attack, emotions are high an important factor for their stability, especially the negative ones, and therefore it is necessarily needed an integrative approach to treatment, taking into account the mental state as a whole especially when they are in a state of psychosocial stress.

**Disclosure of Interest:**

None Declared

